# Impact of postoperative complications on survival after oesophagectomy for oesophageal cancer

**DOI:** 10.1002/bjs5.50264

**Published:** 2020-02-17

**Authors:** J. R. Bundred, A. C. Hollis, R. Evans, J. Hodson, J. L. Whiting, E. A. Griffiths

**Affiliations:** ^1^ College of Medical and Dental Sciences University of Birmingham Birmingham UK; ^2^ Institute of Cancer and Genomic Sciences, College of Medical and Dental Sciences University of Birmingham Birmingham UK; ^3^ Department of Upper Gastrointestinal Surgery University Hospitals Birmingham NHS Foundation Trust Birmingham UK; ^4^ Institute of Translational Medicine University Hospitals Birmingham NHS Foundation Trust Birmingham UK

## Abstract

**Background:**

Recent evidence suggests that complications after oesophagectomy may decrease short‐ and long‐term survival of patients with oesophageal cancer. This study aimed to analyse the impact of complications on survival in a Western cohort.

**Methods:**

Complications after oesophagectomy were recorded for all patients operated on between January 2006 and February 2017, with severity defined using the Clavien–Dindo classification. Associations between complications and overall and recurrence‐free survival were assessed using univariable and multivariable Cox regression models.

**Results:**

Of 430 patients, 292 (67·9 per cent) developed postoperative complications, with 128 (39·8 per cent) classified as Clavien–Dindo grade III or IV. No significant associations were detected between Clavien–Dindo grade and either tumour (T) (*P* = 0·071) or nodal (N) status (*P* = 0·882). There was a significant correlation between Clavien–Dindo grade and ASA fitness grade (*P* = 0·032). In multivariable analysis, overall survival in patients with Clavien–Dindo grade I complications was similar to that in patients with no complications (hazard ratio (HR) 0·97, *P* = 0·915). However, patients with grade II and IV complications had significantly shorter overall survival than those with no complications: HR 1·64 (*P* = 0·007) and 1·74 (*P* = 0·013) respectively.

**Conclusion:**

Increasing severity of complications after oesophagectomy was associated with decreased overall survival. Prevention of complications should improve survival.

## Graphical abstract

Recent evidence suggests that complications following oesophagectomy may decrease short‐ and long‐term survival. In this study, Clavien–Dindo grade II and IV complications decreased overall and recurrence‐free survival in univariable and multivariable Cox regression analyses. Complications may thus be an important prognostic factor, and early intervention to ameliorate their severity may improve both short‐ and long‐term survival.

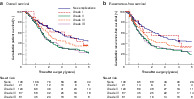

Complication severity influences outcome

## Abstract (da)

**Antecedentes:**

Evidencias recientes sugieren que las complicaciones después de la esofaguectomía pueden reducir la supervivencia a corto y a largo plazo de los pacientes con cáncer de esófago. Este estudio tenía como objetivo analizar el impacto de las complicaciones sobre la supervivencia en una cohorte occidental.

**Métodos:**

Entre enero de 2006 y febrero de 2017, se recogieron las complicaciones tras la esofaguectomía de todos los pacientes, con la gravedad de dichas complicaciones definida según los grados de Clavien. Se evaluaron las asociaciones entre las complicaciones y las supervivencias global y libre de recidiva utilizando modelos de regresión de Cox univariable y multivariable.

**Resultados:**

De 430 pacientes, 292 (68%) desarrollaron complicaciones postoperatorias, con 128 (30%) clasificadas como Clavien‐Dindo grado 3 o 4. No se detectaron asociaciones significativas entre los grados de Clavien‐Dindo y el estadio tumoral (T) o ganglionar (N) (*P* = 0,071, *P* = 0,882). Hubo una correlación significativa entre el grado de Clavien‐Dindo y la puntuación de la *American Society of Anaesthesiologist's* (ASA) (*P* = 0,032). En el análisis multivariable, la supervivencia de los pacientes con complicaciones grado 1 de Clavien‐Dindo era similar a la de aquellos pacientes sin complicaciones (cociente de riesgos instantáneos, *hazard ratio*, HR 0,97, *P* = 0,915). Sin embargo, los pacientes con complicaciones grados 2 y 4 de Clavien‐Dindo presentaron una supervivencia global significativamente más corta en ambos casos, con HRs de 1,64 (*P* = 0,007) y 1,74 (*P* = 0,013) respectivamente, en comparación con los pacientes sin complicaciones.

**Conclusión:**

El aumento de la gravedad de las complicaciones tras una esofaguectomía estaba asociado con una disminución de la supervivencia global. La prevención de las complicaciones debería mejorar la supervivencia.

## Introduction

Despite advances in treatment, oesophageal cancer remains the sixth most common cause of cancer‐related mortality worldwide, with an increasing incidence in the West^1^, ^2^. The mainstay of curative therapy for locoregional oesophageal cancer is oesophagectomy, although this procedure is possible only in selected patients. Even in this subset, resection is associated with significant morbidity and mortality^3^, ^4^. Recent series^5^, ^6^, ^7^, ^8^, ^9^, ^10^ have reported complications in 30–70 per cent of patients. Serious complications, including anastomotic leak, conduit necrosis and pulmonary complications, lead to an increased length of hospital stay and return to theatre, and decreased overall survival^11^, ^12^.

Recent evidence^3^, ^6^, ^13^, ^14^, ^15^, ^16^ also suggests that complications after oesophagectomy may decrease overall and disease‐specific survival in patients with complications that resolve initially. A recent systematic review^3^ found that postoperative complications decreased survival following oesophagectomy. However, before publication of the consensus reporting guidelines on oesophageal complications^11^, a lack of standardization of complication reporting made understanding the relationship between complications and long‐term survival difficult.

This study aimed to identify preoperative and perioperative factors associated with the development of complications, and to examine the effect of complication grade on overall and recurrence‐free survival.

## Methods

All consecutive patients who had oesophagectomy for oesophageal cancer at a tertiary referral centre (Queen Elizabeth Hospital, Birmingham) between January 2006 and February 2017*,* identified from a prospectively developed database, were included in the study. Patients were followed up for a minimum of 28 months to June 2019. Patients with inoperable disease undergoing ‘open and close’ procedures, with no resection, were excluded. The data set included demographic, treatment and pathology details along with complications^17^, ^18^, ^19^, ^20^, ^21^. The study was approved by the Queen Elizabeth Hospital clinical audit department. Ethics committee approval was waived, given the nature of the study. Patients did not receive neoadjuvant chemotherapy when: they declined the option after discussion with a consultant oncologist; they had co‐morbidity for which the risks of chemotherapy were considered to outweigh the advantages (for instance patients with renal impairment or cardiovascular disease); or they had tumour staged before surgery as T2 disease or lower. All patients undergoing oesophagectomy with non‐benign findings on histological examination were included.

### Operations

Over the 11‐year period, oesophagectomies were performed by ten specialist upper gastrointestinal consultant surgeons, or trainees under supervision, and classified as open, minimally invasive or hybrid. The decision regarding surgical approach was at the discretion of the consultant surgeon. Completely minimally invasive procedures were introduced in 2008. Anastomotic techniques included hand‐sewn, circular stapled, OrVil™ (Covidien, Mansfield, MA, USA) and semimechanical anastomoses. Postoperative nutritional support was used routinely via feeding jejunostomy, unless, for technical reasons, nasojejunal feeding or total parenteral nutrition was needed. After surgery, all patients were managed initially in a critical care unit before transfer to standard ward care when considered fit. R1 resections were those in which the tumour was present microscopically within 1 mm of the circumferential, distal or proximal margins, as described by the Royal College of Pathologists^22^; R2 resections were those in which tumour could not be removed completely, leaving macroscopic residual tumour.

### Complications

All postoperative complications were classified according to the standardized definitions proposed by the Esophageal Complications Consensus Group^11^. All complications were graded according to both the Comprehensive Complication Index (CCI) and the Clavien–Dindo classification.

In summary, the Clavien–Dindo system classifies complications as follows. Grade I includes any deviation from the normal postoperative course without the need for pharmacological treatment or surgical, endoscopic or radiological interventions. Acceptable therapeutic regimens are antiemetics, antipyretics, analgesics, diuretics, electrolytes and physiotherapy. This grade also includes wound infections opened at the bedside. Grade II includes any complication requiring pharmacological treatment, not allowed in grade I. Grade III includes any complication requiring surgical, endoscopic or radiological intervention. Grade IV includes single or multiple organ failure, requiring ICU management, and grade V denotes inpatient death.

The CCI produces a score between 1 and 100, allowing for multiple complications, and based on the ranking scale used in the Clavien–Dindo system^23^. Anastomotic leak, chyle leak, conduit necrosis and vocal cord palsy were also graded in severity from I to III, based on the grading proposed by the Esophageal Complications Consensus Group.

### Survival data

Patient survival was calculated from the time of surgery, and censored at the final follow‐up. For analysis of recurrence‐free survival, patients with R2 resections were excluded. Although inpatient death is considered a complication in the Clavien–Dindo grading system (grade V), it was not meaningful to include these patients in the comparisons of long‐term survival by complication grade. As a result, all patients who died as inpatients, and those who died or were lost to follow‐up within 90 days of surgery, were excluded from survival analyses.

### Statistical analysis

Initially, the demographics of the cohort were summarized, using mean(s.d.) and median (i.q.r.) values as appropriate. Patients were then divided into groups based on the highest Clavien–Dindo complication grade: no complication, grades I*–*II and grades III–V. Comparisons between these groups were performed using Jonckheere–Terpstra tests for continuous variables and Kendall's τ for ordinal variables. For nominal variables, the complication grade was compared across categories using the Kruskal–Wallis test.

Postoperative patient survival was compared across complication grades using Kaplan–Meier curves, with univariable Cox regression models to produce hazard ratios (HRs). Multivariable Cox regression models were then produced, to account for the effects of potentially confounding factors, using a backwards stepwise approach to variable selection. All preoperative and intraoperative factors with at least 90 per cent completeness of data were considered initially for inclusion in the models. Before analysis, continuous variables were divided into categories based on the quartiles of the distribution, in order to improve model fit. Factors selected for inclusion by the stepwise procedure were then entered into a new model, to maximize the included sample size, by preventing exclusions owing to missing data for non‐significant factors. As a sensitivity analysis, factors with less than 90 per cent data completeness were then added to the final model, to test whether any of these were significant independent predictors of patient outcomes.

All analyses were performed using IBM SPSS® version 22 (IBM, Armonk, New York, USA), with *P* < 0·050 deemed to be indicative of statistical significance throughout.

## Results

Data were available for a total of 430 patients undergoing surgery between 1 January 2006 and 28 February 2017. The mean(s.d.) age at surgery was 64·9(9·4) years, and the majority of patients were men (79·5 per cent). The majority of patients had preoperative chemotherapy (342 of 430, 79·5 per cent), with the remainder receiving no neoadjuvant therapy. Postoperative histological examination revealed that the majority of the 430 patients had either adenocarcinoma (337, 78·4 per cent) or squamous cell carcinoma (70, 16·3 per cent); the rest had either adenosquamous carcinoma (8, 1·9 per cent) or another malignant cancer (15, 3·5 per cent). Complete patient details including tumour and perioperative/postoperative factors are shown in *Tables* 1, 2, 3.

**Table 1:** Associations between complication grade and patient demographics and co‐morbidity

			Highest Clavien–Dindo grade			
	Total *n* †	Overall	None	I–II	III–V	*P* ‡
**Age at operation (years)** *	430	64·9(9·4)	63·7(10·3)	65·6(8·5)	65·3(9·2)	0·162§
**Male sex**	430	342 (79·5)	109 of 138 (79·0)	120 of 144 (83·3)	113 of 148 (76·4)	0·566
**BMI (kg/m** ^**2**^ **)** *	409	26·4(4·9)	27·1(5·4)	26·2(4·9)	25·9(4·4)	0·139§
**ASA grade**	390		*n* = 117	*n* = 138	*n* = 135	0·032
1		79 (20·3)	25 (21·4)	31 (22·5)	23 (17·0)	
2		207 (53·1)	67 (57·3)	75 (54·3)	65 (48·1)	
3		96 (24·6)	22 (18·8)	31 (22·5)	43 (31·9)	
4		8 (2·1)	3 (2·6)	1 (0·7)	4 (3·0)	
**ECOG performance score**	303		*n* = 93	*n* = 110	*n* = 100	0·041
0		134 (44·2)	48 (52)	51 (46·4)	35 (35·0)	
1		136 (44·9)	33 (35)	52 (47·3)	51 (51·0)	
2		33 (10·9)	12 (13)	7 (6·4)	14 (14·0)	
**Co‐morbidity**			*n* = 125	*n* = 138	*n* = 140	
Ischaemic heart disease	403	53 (13·2)	13 (10·4)	18 (13·0)	22 (15·7)	0·198
Renal impairment	403	4 (1·0)	2 (1·6)	1 (0·7)	1 (0·7)	0·512
Diabetic	403	48 (11·9)	10 (8·0)	18 (13·0)	20 (14·3)	0·109
COPD	403	31 (7·7)	7 (5·6)	10 (7·2)	14 (10·0)	0·178
Previous cancer	403	19 (4·7)	5 (4·0)	4 (2·9)	10 (7·1)	0·242
Significant smoking history	403	60 (14·9)	14 (11·2)	25 (18·1)	21 (15·0)	0·399
Alcohol misuse/heavy drinker	403	9 (2·2)	1 (0·8)	4 (2·9)	4 (2·9)	0·226

Values in parentheses are percentages unless indicated otherwise;

* values are mean(s.d.).

† Number of patients with data available for the stated factor. ECOG, Eastern Cooperative Oncology Group; COPD, chronic obstructive pulmonary disease.

‡ Kendall's τ, except

§ Jonckheere–Terpstra test.

**Table 2:** Associations between complication grade and pathology

			Highest Clavien–Dindo grade			
	Total *n* †	Overall	None	I–II	III–V	*P* ‡
**Type of tumour**	430		*n* = 138	*n* = 144	*n* = 148	0·490§
Adenocarcinoma		337 (78·4)	110 (79·7)	119 (82·6)	108 (73·0)	
Squamous		70 (16·3)	21 (15·2)	17 (11·8)	32 (21·6)	
Adenosquamous		8 (1·9)	2 (1·4)	4 (2·8)	2 (1·4)	
Other		15 (3·5)	5 (3·6)	4 (2·8)	6 (4·1)	
**Tumour location**	392		*n* = 118	*n* = 134	*n* = 140	0·208§
GOJ		236 (60·2)	81 (68·6)	74 (55·2)	81 (57·9)	
Distal		132 (33·7)	30 (25·4)	51 (38·1)	51 (36·4)	
Middle		24 (6·1)	7 (5·9)	9 (6·7)	8 (5·7)	
**pT category**	427		*n* = 135	*n* = 144	*n* = 148	0·071
pT0		19 (4·4)	5 (3·7)	8 (5·6)	6 (4·1)	
pT1		52 (12·2)	13 (9·6)	18 (12·5)	21 (14·2)	
pT2		54 (12·6)	15 (11·1)	14 (9·7)	25 (16·9)	
pT3		277 (64·9)	93 (68·9)	96 (66·7)	88 (59·5)	
pT4		25 (5·9)	9 (6·7)	8 (5·6)	8 (5·4)	
**pN category**	429		*n* = 137	*n* = 144	*n* = 148	0·882
pN0		164 (38·2)	57 (41·6)	46 (31·9)	61 (41·2)	
pN1		186 (43·4)	57 (41·6)	65 (45·1)	64 (43·2)	
pN2		46 (10·7)	13 (9·5)	20 (13·9)	13 (8·8)	
pN3		33 (7·7)	10 (7·3)	13 (9·0)	10 (6·8)	
**pM1 status**	430	9 (2·1)	3 of 138 (2·2)	2 of 144 (1·4)	4 of 148 (2·7)	0·757
**Overall stage**	425		*n* = 134	*n* = 144	*n* = 147	0·428
0		15 (3·5)	4 (3·0)	5 (3·5)	6 (4·1)	
1		74 (17·4)	18 (13·4)	24 (16·7)	32 (21·8)	
2		95 (22·4)	39 (29·1)	25 (17·4)	31 (21·1)	
3		232 (54·6)	70 (52·2)	88 (61·1)	74 (50·3)	
4		9 (2·1)	3 (2·2)	2 (1·4)	4 (2·7)	
**Perineural invasion**	331	105 (31·7)	38 of 114 (33·3)	40 of 115 (34·8)	27 of 102 (26·5)	0·291
**Tumour dimensions (mm)** *						
Length	394	35 (25–45)	35 (25–45)	34 (25–45)	35 (25–48)	0·766¶
Width	382	26 (20–40)	25 (18–40)	25 (20–35)	30 (20–37)	0·774¶
Depth	310	12 (8–15)	12 (8–16)	11 (7–15)	12 (8–16)	0·634¶
Maximum dimension	402	35 (25–50)	36 (25–50)	35 (25–50)	35 (25–50)	0·753¶

Values in parentheses are percentages unless indicated otherwise;

* values are median (i.q.r.).

† Number of patients with data available for the stated factor. GOJ, gastro‐oesophageal junction.

‡ Kendall's τ, except

§ Kruskal–Wallis test and

¶ Jonckheere–Terpstra test.

**Table 3:** Associations between complication grade and intraoperative and postoperative factors

			Highest Clavien–Dindo grade			
	Total *n* ‡	Overall	None	I–II	III–V	*P* #
**Type of operation**	430		*n* = 138	*n* = 144	*n* = 148	0·436**
Hybrid		223 (51·9)	71 (51·4)	70 (48·6)	82 (55·4)	
MIO		101 (23·5)	26 (18·8)	43 (29·9)	32 (21·6)	
Open		106 (24·7)	41 (29·7)	31 (21·5)	34 (23·0)	
**Total no. of LNs** *	429	30·3(11·5)	31·4(11·6)	29·5(11·1)	30·0(11·8)	0·239††
**No. of LNs involved** †	429	1 (0–4)	1 (0–3)	2 (0–4)	1 (0–4)	0·778††
**% of LNs involved** †	429	4 (0–13)	4 (0–10)	7 (0–16)	4 (0–13)	0·783††
**Margins involved**						
Proximal	428	11 (2·6)	5 of 138 (3·6)	4 of 143 (2·8)	2 of 147 (1·4)	0·211
Distal	428	5 (1·2)	0 of 138 (0)	2 of 143 (1·4)	3 of 147 (2·0)	0·089
Circumferential	420	140 (33·3)	40 of 132 (30·3)	54 of 141 (38·3)	46 of 147 (31·3)	0·916
**R status**	423		*n* = 133	*n* = 143	*n* = 147	0·682
R0		257 (60·8)	82 (61·7)	81 (56·6)	94 (63·9)	
R1		152 (35·9)	48 (36·1)	55 (38·5)	49 (33·3)	
R2		14 (3·3)	3 (2·3)	7 (4·9)	4 (2·7)	
**Total intraoperative/postoperative blood loss (units)**	430		*n* = 138	*n* = 144	*n* = 148	< 0·001
0		332 (77·2)	116 (84·1)	122 (84·7)	94 (63·5)	
1–2		78 (18·1)	19 (13·8)	15 (10·4)	44 (29·7)	
≥ 3		20 (4·7)	3 (2·2)	7 (4·9)	10 (6·8)	
**CRP (mg/l) on day 4** *, §						
Actual	285	217·3(94·8)	196·5(84·4)	200·5(87·6)	247·1(101·0)	< 0·001††
LMCF	419	207·5(93·5)	188·8(81·5)	196·9(87·5)	236·9(103·6)	< 0·001††
**Albumin (g/l) on day 4** *, §						
Actual	319	26·6(4·5)	27·7(3·7)	27·6(4·6)	24·6(4·3)	< 0·001††
LMCF	423	26·8(4·4)	27·9(3·8)	27·6(4·4)	24·9(4·5)	< 0·001††
**WCC (× 10** ^**9**^ **/l) on day 4** *, §						
Actual	351	9·5(3·5)	9·0(3·0)	9·3(2·9)	10·1(4·2)	0·050††
LMCF	424	9·8(3·7)	9·6(3·7)	9·5(3·2)	10·3(4·2)	0·123††
**Neoadjuvant chemotherapy**	430	342 (79·5)	112 of 138 (81·2)	113 of 144 (78·5)	117 of 148 (79·1)	0·665
**Return to theatre**	430	90 (20·9)	2 of 138 (1·4)	1 of 144 (0·7)	87 of 148 (58·8)	< 0·001
**Return to ICU**	426	86 (20·2)	0 of 137 (0)	8 of 143 (5·6)	78 of 146 (53·4)	< 0·001
**Total length of hospital stay (days)** †	430	16 (11–25)	12 (9–15)	16 (11–20)	27 (17–43)	< 0·001††
**Total length of ICU stay (days)** †	430	5 (2–10)	3 (2–4)	4 (2–7)	11 (5–19)	< 0·001††

Values in parentheses are percentages unless indicated otherwise;

* values are mean(s.d.) and

† median (i.q.r.).

‡ Number of patients with data available for the stated factor.

§ ‘Actual’ gives measurements recorded on day 4, whereas last measure carried forward (LCMF) fills in missing data using the most recent value obtained before day 4, where possible. MIO, minimally invasive operation; LN, lymph node; CRP, C‐reactive protein; WCC, white cell count.

# Kendall's τ, except

** Kruskal–Wallis test and

†† Jonckheere–Terpstra test.

### Complications

**Figure 1:**
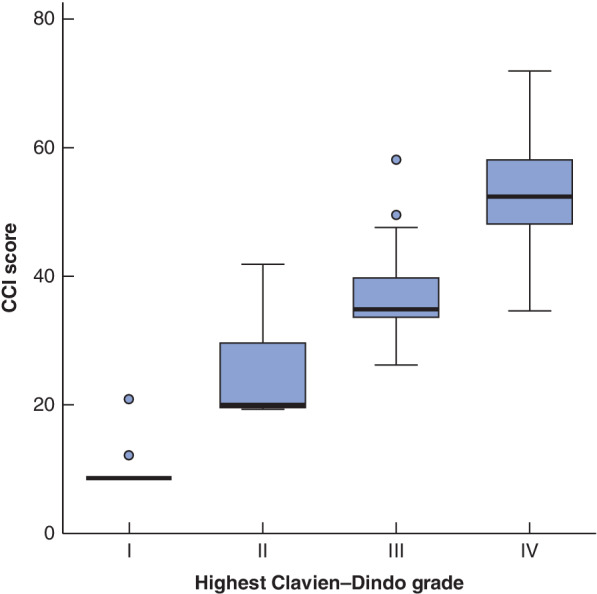
Box‐and‐whisker plot of the association between Clavien–Dindo grade and Comprehensive Complication Index score in patients with complications The plot is based on 242 patients, after excluding 138 with no complications, 20 who died after surgery (Clavien–Dindo grade V/Comprehensive Complication Index (CCI) score 100) and 30 with no CCI score recorded. Median values, interquartile ranges and ranges (excluding outliers) are denoted by horizontal bars, boxes and error bars respectively (*r*_s_ = 0·91, *P* < 0·001).

A total of 292 patients (67·9 per cent) developed postoperative complications, the most common being infection (49·8 per cent), pulmonary (46·5 per cent) and gastrointestinal (24·9 per cent) related (*Table* 4). For 144 patients (33·5 per cent), the highest recorded Clavien–Dindo grade was I or II, and 128 patients (29·8 per cent) had grade III or IV as the highest recorded grade. The remaining 20 patients died in hospital (Clavien–Dindo grade V). The CCI score was available for only 383 patients (median 21 (i.q.r. 0–40)). The CCI score and Clavien–Dindo grade were highly correlated (*r*_s_ = 0·91, *P* < 0·001) (*Fig*. *1*), so only the Clavien–Dindo system was used in subsequent analyses.

**Table 4:** Postoperative complications

	Total *n* ‡	No. of patients*
**Highest Clavien–Dindo grade**	430	
No complications		138 (32·1)
I		21 (4·9)
II		123 (28·6)
III		67 (15·6)
IV		61 (14·2)
V (death)		20 (4·7)
**CCI score** †	383	21 (0–40)
**Type of complication**	430	
None		138 (32·1)
Medical only		47 (10·9)
Surgical only		55 (12·8)
Both medical and surgical		190 (44·2)
**Infective complication**	430	214 (49·8)
**Pulmonary complication**	430	200 (46·5)
**Gastrointestinal complication**	430	107 (24·9)
**Cardiac complication**	430	91 (21·2)
**Neurological/psychiatric complication**	430	21 (4·9)
**Wound/diaphragm complication**	430	18 (4·2)
**Urological complication**	430	14 (3·3)
**Thromboembolic complication**	430	6 (1·4)
**Anastomotic leak grade**	430	
No leak		363 (84·4)
I		14 (3·3)
II		18 (4·2)
III		35 (8·1)
**Conduit necrosis grade**	430	
No necrosis		416 (96·7)
I		1 (0·2)
II		5 (1·2)
III		8 (1·9)
**Chyle leak grade**	430	
No leak		399 (92·8)
I		19 (4·4)
II		3 (0·7)
III		9 (2·1)
**Vocal cord palsy grade**	430	
No palsy		417 (97·0)
I		8 (1·9)
II		4 (0·9)
III		1 (0·2)

* With percentages in parentheses unless indicated otherwise;

† values are median (i.q.r.).

‡ Number of patients with data available for the stated factor. CCI, Comprehensive Complication Index.

### Factors associated with complication grade

Patients who developed complications were generally similar to those without complications with regard to demographic (*Table* 1) and tumour‐related (*Table* 2) factors. The only significant differences were in the ASA grade (*P* = 0·032) and ECOG score (*P* = 0·041), both of which increased with complication grade. Of the postoperative factors considered (*Table* 3), patients with higher‐grade complications required significantly more units of blood, and also had significantly higher C‐reactive protein (CRP) and lower albumin levels on postoperative day 4 (all *P* < 0·001). Patients with Clavien–Dindo grade III–V complications were significantly more likely than those with either no or minor complications to return to both theatre (87 of 148 (58·8 per cent) *versus* 3 of 282 (1·1 per cent) respectively; *P* < 0·001) and ICU (78 of 146 (53·4 per cent) *versus* 8 of 280 (2·9 per cent); *P* < 0·001). Consequently, the lengths of ITU and total hospital stay were significantly longer for patients with Clavien–Dindo grade III–V complications (both *P* < 0·001).

### Survival by complication grade

After exclusion of the 20 patients who died in hospital, a further 14 patients who were lost to follow‐up less than 90 days after surgery were also excluded. The remaining 396 patients had a median follow‐up of 23·6 (i.q.r. 13·5–50·7) months, during which there were 258 deaths, giving Kaplan–Meier‐estimated overall survival rates of 81·4, 44·5 and 33·1 at 1, 3 and 5 years respectively.

**Figure 2:**
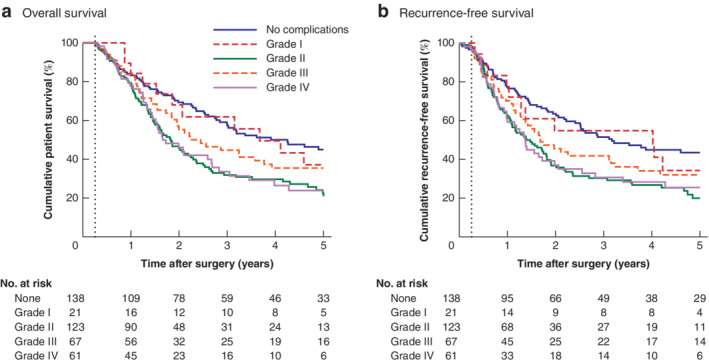
**Kaplan–Meier analysis of postoperative survival by highest Clavien–Dindo grade** **a** Overall and **b** recurrence‐free survival. Patients who died or were lost to follow‐up within 90 days (dotted line) were excluded. The plot of recurrence‐free survival additionally excludes 18 patients with R2 or unknown resection status.

Patient survival was found to differ significantly by Clavien–Dindo grade (*P* < 0·001) (*Table* 5 and *Fig*. *2*). Patients with grade I complications had similar overall survival to those with no complications (median 44 *versus* 51 months respectively) (HR 1·28, *P* = 0·419). However, higher‐grade complications were associated with significantly shorter survival: median survival 22, 27 and 20 months for Clavien–Dindo grades II, III and IV respectively. Analysis of recurrence‐free survival gave similar results.

**Table 5:** Survival outcomes by Clavien–Dindo grade

	Overall survival				Recurrence‐free survival†			
	*n*	Median (months)‡	Hazard ratio§	*P* §	*n*	Median (months)‡	Hazard ratio§	*P* §
**Highest Clavien–Dindo grade**				< 0·001				< 0·001
No complications	133	51·0	1·00 (reference)		126	38·1	1·00 (reference)	
I	19	44·1	1·28 (0·71, 2·31)	0·419	18	48·3	1·42 (0·79, 2·59)	0·245
II	118	21·6	2·01 (1·46, 2·75)	< 0·001	113	17·7	1·97 (1·43, 2·73)	< 0·001
III	67	26·7	1·40 (0·96, 2·04)	0·081	64	20·2	1·50 (1·03, 2·20)	0·036
IV	59	19·9	1·97 (1·34, 2·90)	< 0·001	57	16·5	1·95 (1·32, 2·88)	< 0·001

Values in parentheses are 95 per cent confidence intervals. Patients who died or were lost to follow‐up within 90 days were excluded;

† additionally excludes 18 patients with R2 or unknown resection status.

‡ Kaplan–Meier estimates;

§ univariable Cox regression models.

Multivariable survival analysis included all of the factors in *Tables* 1, 2, 3, alongside the Clavien–Dindo complication grade, except for total number of units of blood, return to theatre/ICU and lengths of hospital/ITU stay, as these were consequences of complications. Overall stage and number of lymph nodes involved were also excluded, as these were highly correlated with TNM staging and proportion of lymph nodes involved respectively. Finally, the ECOG score, tumour depth, length and width, and the presence of perineural invasion were initially excluded, as the amount of missing data exceeded 10 per cent.

For patient survival, the resulting model (*Table* 6) found increasing proportions of lymph nodes involved, unsuspected M1 disease on postoperative histology and low albumin levels on postoperative day 4 to be the strongest predictors of poor prognosis (all *P* < 0·001). After accounting for these and the other factors in the model, the association between Clavien–Dindo complication grade and overall survival remained significant (*P* = 0·029). The results were similar to those observed in the univariable analysis, with Clavien–Dindo grade I complications not found to influence survival significantly (HR 0·97, *P* = 0·915), but grade II (HR 1·64, *P* = 0·007) and grade IV (HR 1·74, *P* = 0·013) being significant independent predictors of poorer overall survival. However, overall survival after Clavien–Dindo grade III complications was not significantly different to that after no complications (HR 1·24, *P* = 0·336). Analysis of recurrence‐free survival returned similar results. As a sensitivity analysis, the factors excluded owing to excessive missing data were then added to the final models; none was found to be a significant independent predictor of either overall or recurrence‐free survival.

**Table 6:** Multivariable analysis of survival outcomes

	Overall survival		Recurrence‐free survival	
	Hazard ratio	*P*	Hazard ratio	*P*
**Highest Clavien–Dindo grade**		0·029		0·041
No complications	1·00 (reference)		1·00 (reference)	
I	0·97 (0·51, 1·83)	0·915	1·37 (0·72, 2·62)	0·341
II	1·64 (1·14, 2·35)	0·007	1·63 (1·13, 2·35)	0·009
III	1·24 (0·80, 1·93)	0·336	1·22 (0·78, 1·90)	0·376
IV	1·74 (1·13, 2·70)	0·013	1·82 (1·17, 2·82)	0·007
**ASA grade**		0·035		0·030
I	1·00 (reference)		1·00 (reference)	
II	0·64 (0·45, 0·90)	0·011	0·64 (0·45, 0·91)	0·013
III	0·91 (0·62, 1·35)	0·655	0·88 (0·59, 1·30)	0·526
IV	0·56 (0·21, 1·47)	0·241	0·42 (0·16, 1·11)	0·080
**pM1 status**	5·34 (2·36, 12·05)	< 0·001	2·97 (1·13, 7·80)	0·027
**% of lymph nodes involved**		< 0·001		< 0·001
0	1·00 (reference)		1·00 (reference)	
1–5	1·54 (0·97, 2·45)	0·065	1·68 (1·05, 2·69)	0·030
6–15	2·98 (2·04, 4·35)	< 0·001	2·90 (1·98, 4·24)	< 0·001
> 15	4·93 (3·33, 7·30)	< 0·001	5·18 (3·44, 7·79)	< 0·001
**R1 status**	n.s.		1·33 (0·99, 1·79)	0·057
**Distal margin involved**	0·27 (0·08, 0·88)	0·029	n.s.	
**Albumin (g/l) on day 4** *		< 0·001		0·015
< 24	1·00 (reference)		1·00 (reference)	
24–26	0·60 (0·40, 0·91)	0·015	0·75 (0·50, 1·13)	0·171
27–29	0·50 (0·34, 0·75)	< 0·001	0·61 (0·41, 0·90)	0·012
≥ 30	0·45 (0·30, 0·67)	< 0·001	0·54 (0·37, 0·81)	0·003

Values in parentheses are 95 per cent confidence intervals. Cox regression models were used with a backwards stepwise approach to variable selection; all factors from *Tables* 1, 2, 3 with at least 90 per cent completeness of data were considered for inclusion in the models, with the exception of total units of blood, return to theatre/ICU and length of hospital/ICU stay. The backwards stepwise approach was then repeated for the factors selected by the initial model, to maximize the included sample size. The final models were based on 339 patients (218 events) for overall survival and 331 patients (214 events) for recurrence‐free survival. The latter model excluded patients with R2 resection status.

* A last measure carried forwards approach was used to fill in missing data using the most recent measurement obtained before day 4, where possible. n.s., Not selected for inclusion in the final model by the stepwise procedure.

## Discussion

Clavien–Dindo grade II and IV complications were independently associated with significantly shorter overall and recurrence‐free postoperative survival in patients undergoing oesophagectomy for oesophageal cancer.

There are several possible reasons for the association between complications and poorer prognosis. Complications may lead to increased inflammation, affecting the immune system of patients after surgery and leading to increased production of proinflammatory cytokines such as interleukin (IL) 6 and IL‐8^24^. This has been hypothesized to decrease the ability of the immune system to repress tumour recurrence. Previous reports^25^, ^26^ have similarly implicated a variety of inflammatory mediators in cancer recurrence and progression. Inflammatory pathways acting within the tumour microenvironment are also known to contribute to tumour growth, and to promote both survival and the growth of micrometastases, locally and at distant sites^27^. The finding that there was a correlation between the Clavien–Dindo grade and both CRP and albumin levels on postoperative day 4 could be interpreted as supporting evidence for this hypothesis. Preoperative albumin concentration is a well known prognostic marker; however, fewer studies have assessed the prognostic value of postoperative albumin^18^. Although a decrease in albumin after surgery probably reflects the systemic inflammatory response to surgery and was associated with higher‐grade complications, decreased albumin levels have been shown to be associated with other adverse outcomes^28^, ^29^, ^30^ and were independently predictive of survival in multivariable analysis in the present study.

This study included a large cohort of patients with oesophageal cancer, with almost complete follow‐up, and loss to follow‐up was accounted for in the modelling. The analysis of individual complication grades builds on the results of a recent systematic review^3^, allowing a more in‐depth picture of how complications affect overall survival and disease recurrence. Similar rates of complications and anastomotic leak have been reported in other recent Western cohorts^31^, ^32^, ^33^, ^34^. It is worth noting that the present series included learning curves for minimally invasive oesophagectomy^35^, and a possible increase in leak rate as a result of the vascular endothelial growth factor inhibitors used in patients who took part in the ST03 trial^36^.

Limitations of the study include the length of the study period, which included the introduction of minimally invasive oesophagectomy with a number of different techniques. Neoadjuvant therapies changed over the study period, with initially the MAGIC^37^ and then the OE02^38^, OE05^39^ and ST03^36^ trials ushering in an era of increasingly potent perioperative chemotherapy.

This study specifically analysed the impact of individual Clavien–Dindo grades on survival. Other studies generally combine grade III and IV complications, owing to sample size, and many have not analysed grade I or II complications at all^3^, ^6^, ^7^, ^8^, ^9^, ^40^. In the present study, Clavien–Dindo grade II and IV complications were independently associated with decreased postoperative overall and recurrence‐free survival. However, although grade III complications were significantly associated with overall survival in univariable analysis, there was no significant association in multivariable analysis. This may be due to the relatively small number of patients with grade III complications and therefore an insufficient sample size for multivariable significance. An alternative explanation is that the early and aggressive treatment of complications with radiological, endoscopic or surgical methods could lead to a shorter period of physiological stress for patients, and thereby affect oncological outcomes. ‘Failure to rescue’ is a well known phenomenon^41^, ^42^, impacting on short‐term survival after oesophagectomy, and possibly explaining differences in mortality between high‐ and low‐volume centres. Failure to identify complications early and treat patients may also contribute to a long‐term impact of complications on patient survival^41^, ^42^, ^43^.

As complications were independently predictive of survival, regardless of tumour stage or grade, it follows that reducing the likelihood of complications may improve survival. Recent studies have focused on prehabilitation^44^, enhanced recovery after surgery^45^, intensive postoperative physiotherapy and incentive spirometry^46^, with mixed findings. Additionally, minimally invasive and hybrid oesophagectomy techniques appear to reduce pulmonary complications^47^, ^48^. In the present study, no association existed between operative technique and complications, although the impact of learning curves merits consideration^35^. Techniques that might decrease anastomotic leak rates, such as omental wrapping^49^ and indocyanine green^50^ assessment, may impact on survival in the future.

## Acknowledgements

J.R.B. and A.C.H. contributed equally to this work.

The authors thank all consultant upper gastrointestinal surgeons from UHB and the Upper GI MDT for allowing their patients to be included in the study.

This study was completed with funding from the Queen Elizabeth Hospital Birmingham Charity (Upper Gastrointestinal Fund) and the Upper G.I. Blues charity, Sandwell.

*Disclosure*: The authors declare no conflict of interest.
